# Effects of Pre-Processing Short-Term Hot-Water Treatments on Quality and Shelf Life of Fresh-Cut Apple Slices

**DOI:** 10.3390/foods8120653

**Published:** 2019-12-06

**Authors:** Guido Rux, Efecan Efe, Christian Ulrichs, Susanne Huyskens-Keil, Karin Hassenberg, Werner B. Herppich

**Affiliations:** 1 Department of Horticultural Engineering, Leibniz Institute for Agricultural Engineering and Bioeconomy (ATB), Max-Eyth-Allee 100, 14469 Potsdam, Germany; grux@atb-potsdam.de (G.R.); efeefeca@cms.hu-berlin.de (E.E.); khassenberg@atb-potsdam.de (K.H.); 2 Thaer-Institute of Agricultural and Horticultural Sciences, Division Urban Plant Ecophysiology, Section Quality Dynamics/Postharvest Physiology, Humboldt-Universität zu Berlin, Lentzeallee 55/57, 14195 Berlin, Germany; christian.ulrichs@hu-berlin.de (C.U.); susanne.huyskens@hu-berlin.de (S.H.-K.)

**Keywords:** minimal processing, sugar syrup immersion, microbial analyses, chemical prevention, ready-to eat fruit salads

## Abstract

Processing, especially cutting, reduces the shelf life of fruits. In practice, fresh-cut fruit salads are, therefore, often sold immersed in sugar syrups to increase shelf life. Pre-processing short-term hot-water treatments (sHWT) may further extend the shelf life of fresh-cuts by effectively reducing microbial contaminations before cutting. In this study, fresh-cut ‘Braeburn’ apples, a major component of fruit salads, were short-term (30 s) hot water-treated (55 °C or 65 °C), partially treated with a commercial anti-browning solution (ascorbic/citric acid) after cutting and, thereafter, stored immersed in sugar syrup. To, for the first time, comprehensively and comparatively evaluate the currently unexplored positive or negative effects of these treatments on fruit quality and shelf life, relevant parameters were analyzed at defined intervals during storage at 4 °C for up to 13 days. Compared to acid pre-treated controls, sHWT significantly reduced the microbial loads of apple slices but did not affect their quality during the 5 day-standard shelf life period of fresh-cuts. Yeasts were most critical for shelf life of fresh-cut apples immersed in sugar syrup. The combination of sHWT and post-processing acid treatment did not further improve quality or extend shelf life. Although sHWT could not extend potential maximum shelf life beyond 10 d, results highlighted the potentials of this technique to replace pre-processing chemical treatments and, thus, to save valuable resources.

## 1. Introduction

Processing of fresh-cut fruit salads involves sorting, cleaning, washing, peeling, deseeding/coring and cutting of fruit. Especially for apples, which are often a major component of fruit salads, cutting [1] pronouncedly reduces the shelf life due to increased water losses [2,3], surface browning and accelerated microbial spoilage [4,5]. To optimize the shelf life of fresh-cut fruit, various postharvest treatments are used, alone and in combination [6], such as modified atmosphere and humidity packaging [7,8], edible coatings [9], thermal [10] and plasma [11] treatments, UV-C irradiation combined with modified atmosphere packaging (MAP) [12] or the application of organic acid solutions and sugar syrups [13]. In practice, the latter is in particular used for bulk purchaser [14] to extend the common maximum shelf life for fresh-cut fruit salads of 5 days [15] to up to 10 days.

Despite reports on the effects of storage in sugar syrup on ethylene metabolism [13] or chlorophyll degradation of fresh-cut fruit [16], the knowledge on the impact of sugar solution on the physiological properties of immersed fresh-cuts is still limited. Investigating the effects of complete immersion of apple slices in sugar solutions of different concentrations on various physiological and quality aspects, Rux et al. [6] indicated that concentrations between 13–20% effectively prevented browning and showed best potentials for storage of fresh-cut fruit. The authors also reported that microbial spoilage was the main factor limiting shelf life of the apple slices. Consequently, careful removal of microorganisms, adherent to fruit skin [17] is essential to avoid microbiological contamination and cross-contaminations [18].

Short-term (15 s up to few min) hot-water treatments (sHWT) at relatively high temperatures (40–80 °C) effectively reduce microbial contamination [19] and insect infestations, and maintain storage quality of apples and other fruits [18,20,21]. In addition, sHWT is relatively inexpensive, easy to use and gentle, and needs no chemicals. Therefore, sHWT is particularly suitable for organic production [22,23].

For fruit salad production, sHWT needs to be optimized to prevent fruit injury and reduction of produce quality, and to guarantee the commercially required shelf life of 10 days. Although systematic studies on heat impacts on structure and function of fruit epidermal tissue are mainly restricted to long-term non-aqueous heat treatments at moderate temperatures [24,25], Kabelitz and Hassenberg [18] evaluated the suitability of sHWT for pre-processing of apples for fresh-cut fruit salad production. In addition, Kabelitz et al. [26] investigated sHWT effects on the commodity surface tissue and heat transfer dynamics in “intact” apples. To the authors’ best knowledge, the potential implications of sHWT on the physiological quality parameters of apple slices have not been studied yet under semi-practical conditions during and beyond the maximum shelf life periods (10 days and 13 days, respectively).

Thus, this study aims to evaluate the effects of short-term (30 s) HW-treatment (at 55 °C or 65 °C) on important physiological parameters of fresh-cut ‘Braeburn’ (commercially often used in fresh-cut fruit salads) apple slices, stored immersed in sugar syrup in pails for up to 13 days. sHW treatment conditions were chosen following Kabelitz et al. [26], who showed that treatments at 55 °C optimally maintained fruit quality and efficiently reduced microbial loads, while those at 65 °C were optimal for pathogen inactivation. To simulate practical condition as close as possible, slices were partially treated with a commercial ascorbic/citric acid solution to prevent browning of surface after cutting. At defined intervals, samples were taken to analyze important quality parameters. Care was taken so that all treatments and processes reflect current practical use as close as possible.

For the first time, the currently unexplored positive or negative effects of sHWT and/or of post-processing acid treatment both combined with sugar syrup-storage on fruit quality and shelf life were comprehensively and comparatively evaluate on fresh-cut apple slices. The results of the presented study will allow assessing whether sHWT could potentially supplement or replace post-processing chemical treatments for improving quality maintenance and shelf life, the latter beyond the current maximum shelf life period of 10 days.

## 2. Materials and Methods

### 2.1. Material

Fresh mature ‘Braeburn’ apples (*Malus domestica* Borkh.) were obtained from a commercial fresh-cut salad producer (mirontell fein and frisch AG, Großbeeren, Germany) and transported to the Department of Horticultural Engineering (Leibniz Institute for Agricultural Engineering and Bioeconomy, Potsdam, Germany). Undamaged apples of uniform size were selected and stored at 4 °C and 95% relative humidity for up to 3 days until experiments. Average initial mass (*n* = 20) and dry matter content (*n* = 6) were 150.7 ± 5.1 g and 17.7 ± 1.1 g per 100 g fresh mass (FM), respectively.

### 2.2. Pre-Processing Short-Term Hot-Water Treatment

Following Kabelitz et al. [26], a batch of 4 cooled apples (in total, 16 fruit per treatment) was either water-washed (controls) at room temperature (approx. 20 °C) or short-term hot-water treated at 55 °C or 65 °C for 30 s in a GFL 1086 water bath (Gesellschaft für Labortechnik mbH, Burgwedel, Germany) before cutting. During treatments, apples were kept submerged in water using a stainless steel plate. Before each additional treatment, the water temperature was readjusted and the water replaced after every second washing.

### 2.3. Fresh-Cut Preparation and Sampling

Closely following practical treatments, apples were further processed under hygienic conditions at 4 °C in a cooling room. Apples were halved equatorially, and each half-segment was cored and equally cut into 16 pieces using a Parti apple cutter (Gefu GmbH, Eslohe, Germany).

For the additional anti-browning treatments, the apple slices of some batches were immersed in ascorbic/citric acid solution (40 g ascorbic acid and 20 g citric acid in 1 L deionized water) for 5 min (Table 1), as it is currently applied in practice (mirontell fein and frisch AG, personal communication). Apple slices of each batch were carefully mixed to ensure homogeneous distribution and placed in a commercial 840 mL plastic pail (approx. 48 with ca. 230 g), with three pails for each sampling day as replicates, yielding a total of 12 pails per treatment.

All pails were filled with 450 mL sugar syrup (200 g L^−1^ invert sugar syrup; 72.7%; Hanseatische Zuckerraffinerie GmbH and Co. KG, Hamburg, Germany) and 10 g L^−1^ OBSTSERVAL HC-2 (Konserval, Pharmacon Lebensmittelzusätze GmbH, Trittau, Germany) browning inhibitor (aqueous solution of ascorbic acid, sodium ascorbate and citric acid), tightly closed and stored at 4 °C for up to 13 d. On days 5, 10 and 13, three pails were opened and apple slices or aliquots of the syrup removed for further analyses. Days 5, 10 and 13 represent the common shelf life of fresh-cut fruit salads, the shelf life given by the producer for storage in sugar syrup and the shelf life potentially pro-longed by sHWT, respectively.

### 2.4. Color Measurement

For each treatment sample, both peel surface and tissue color parameters (CIE L*, a*, and b*) of 10 apple slices per treatment were measured with a CM-2600d spectrophotometer (Konica Minolta Sensing Inc., Tokyo, Japan), calibrated against white and black tiles. The browning index, BI [27], was calculated as:

(1) $$\mathrm{BI}=\frac{\left(100\left(x\;-\;0.31\right)\right)}{0.17}\;x=\frac{\left(a\;+\;1.75L\right)}{\left(5.645L\;+\;a\;-\;3.012b\right)}$$

### 2.5. Tissue Strength

For each sample day, tissue strength of 10 apple slices per treatment was determined with a texture analyzer (TA-XT Plus, Stable Micro Systems, Surrey, UK), fitted with a SMS-P/4 cylinder probe. Tissue strength was measured as maximum compression force (N) at 8 mm indentation (speed for test and post-test; 1 and 10 mm s^−1^, respectively).

### 2.6. Total Soluble Solids, Total Titratable Acidity and Vitamin C Contents

Tissue juice was extracted from 10 apple slices per treatments using a garlic press and the juice centrifuged at 14500 rpm for 2 min using MiniSpin Plus (Eppendorf AG, Hamburg, Germany). The total soluble solids content (TSS; °Brix) was measured from aliquots with a DR301-95 electronic refractometer (Krüss Optronic, Hamburg, Germany), while total titratable acidity (TA) was obtained with an automated T50M Titrator (with Rondo 20 sample changer, Mettler Toledo, Gießen, Germany) by titration with 0.1 mol L^−1^ NaOH to pH 8.2. Vitamin C contents (mg per 100 g FM) were determined with a Reflectoquant^®^ test kit (Merck, Darmstadt, Germany).

### 2.7. Microbial Analysis

For microbial analyses, 6 apple slices and approx. 10 mL sugar syrup was removed from each pail. Using the total plate count method, samples were evaluated in duplicate (*n* = 2) either on plate count agar (PCA; Carl Roth GmbH and Co. KG, Karlsruhe, Germany) for total aerobic mesophilic bacterial counts or on rose bengal chloramphenicol agar (RBCA; Carl Roth GmbH and Co. KG) for yeast and mold counts, respectively. Apple samples (10 g) or syrup were transferred into sterile stomacher bags filled with 90 mL buffered peptone water and homogenized with a BagMixer^®^ 400CC^®^ lab blender (Interscience, Saint Nom, France) at speed 4 (10 strokes s^−1^) for 2 min. Thereafter, the samples were serial diluted by adding 30 µL of each diluent into 270 µL of peptone salt solution in Rotilabo^®^-microtest plates (96er U-profile, Carl Roth GmbH and Co KG) and 100 µL from each dilution were pour-plated on respective growth media. Aerobic mesophilic bacterial counts were enumerated after 3 days at 30 °C, whereas yeast and mold were counted after 7 days at 25 °C, and results expressed as colony forming unit per g (CFU g^−1^).

### 2.8. Statistical Analysis

For each sample day and treatment, three pails were used as replicates. For color and tissue strength evaluations, 10 individual apple slices from each pail were measured and their results averaged (*n* = 3). Similarly, for TSS, TA and vitamin C (in duplicate) analyses, 10 apple slices from each pail were juiced, measured and their results averaged (*n* = 3). Microbial loads were determined using 6 homogenized apple slices from each pail, which were analyzed in duplicate and their results averaged (*n* = 3). All data were statistically analyzed (ANOVA) using WinSTAT (R. Fitch Software, Staufen, Germany) and presented as means ± standard deviation (SD). Duncan’s multiple range test (*p* < 0.05) was used to analyze the significance of differences between means.

## 3. Results

### 3.1. Color Evaluation

Neither treatment conditions nor storage time clearly affected any color parameter of both fruit tissue (Table 2) and peel (Table 3). Variations of L*, a*, b* or BI were small and did not reflect any clear trend. Nevertheless, lightness, L*, of stored tissue samples was significantly reduced irrespective of treatments, if compared to unprocessed fruit. Among sHWT samples, differences between peel color parameters were not significant.

### 3.2. Tissue Strength

**Figure 1:**
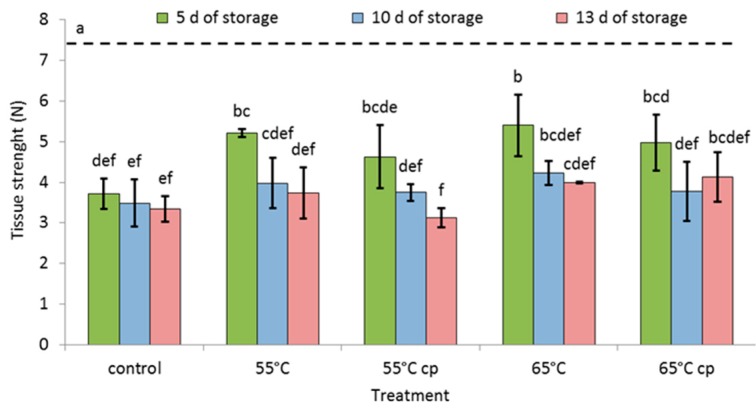
Tissue strength of short-term hot-water treated and untreated fresh-cut apple slices, stored at 4 °C in sugar syrup for up to 13 days compared to that of fresh fruit (dotted line). Given are means ± standard deviation (*n* = 3). Different letters indicate significant differences between means (*p* < 0.05).

The mean initial tissue strength of freshly cut apples slices was 7.4 ± 1.4 N; it significantly and pronouncedly (>25%) declined in all samples irrespective of the treatments during storage (Figure 1). Softening, however, was largest in controls, where tissue strength declined to 3.7 N, i.e., by more than 50% even during the initial 5 days of storage. Compared to controls, all sHWT-samples without additional application of acid solutions had significantly stronger tissue at day 5. Irrespective of treatments, tissue strength continued to decline by 10–32% in all samples with further storage (10 and 13 days).

### 3.3. Total Soluble Solids, Total Titratable Acidity and Vitamin C

**Figure 2:**
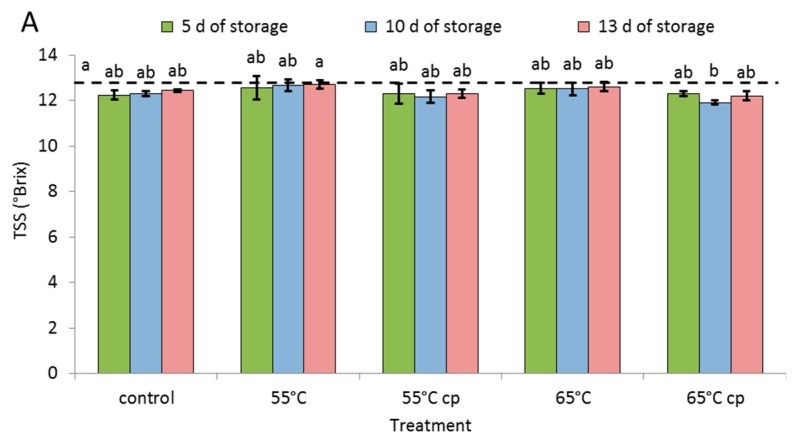

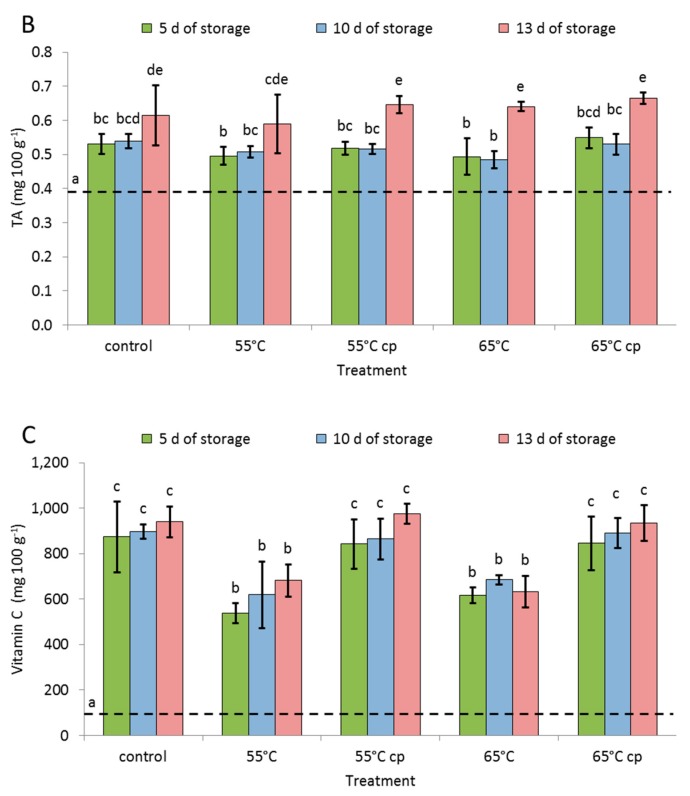
(**A**) Total soluble solids (TSS), (**B**) total titratable acidity (TA) and (**C**) vitamin C content of short-term hot-water treated and untreated fresh-cut apple slices stored at 4 °C in sugar syrup for up to 13 days compare to initial values measured in fresh fruit (dotted line). Given are means ± standard deviation (*n* = 3). Different letters indicate significant differences between means (*p* < 0.05).

Irrespective of the treatments, the initial TSS of fresh apple slices (12.7 °Brix) did only marginally and insignificantly decrease during storage (Figure 2A). Exclusively TSS of samples treated at 65 °C with additional acid treatment (65 cp) slightly declined to 11.9 °Brix at day 10.

In all samples, total titratable acidity (TA) increased during storage, starting from an initial TA of 0.39 mg 100 g^−1^ in fresh apples (Figure 2B). In particular during initial and late storage, the rise in TA content was significant in most samples. During the entire storage, TA was not significantly different in the apple slices, irrespective of the treatments.

The initial vitamin C content of fresh apples (76 mg 100 g^−1^) drastically increased during initial storage, in particular in samples additionally treated with acid solution (Figure 2C). In the latter samples, the additional application of vitamin C at high concentrations (i.e., 40g L^−1^) to the freshly cut slices significantly increased their acid content compared to apples slices without this post-processing treatment (vitamin C content in sugar syrup < 10 g L^−1^). In all samples, vitamin C contents tended to increase during further storage although at lower rates due to the reduced concentration gradients.

### 3.4. Microbial Analysis

**Figure 3:**
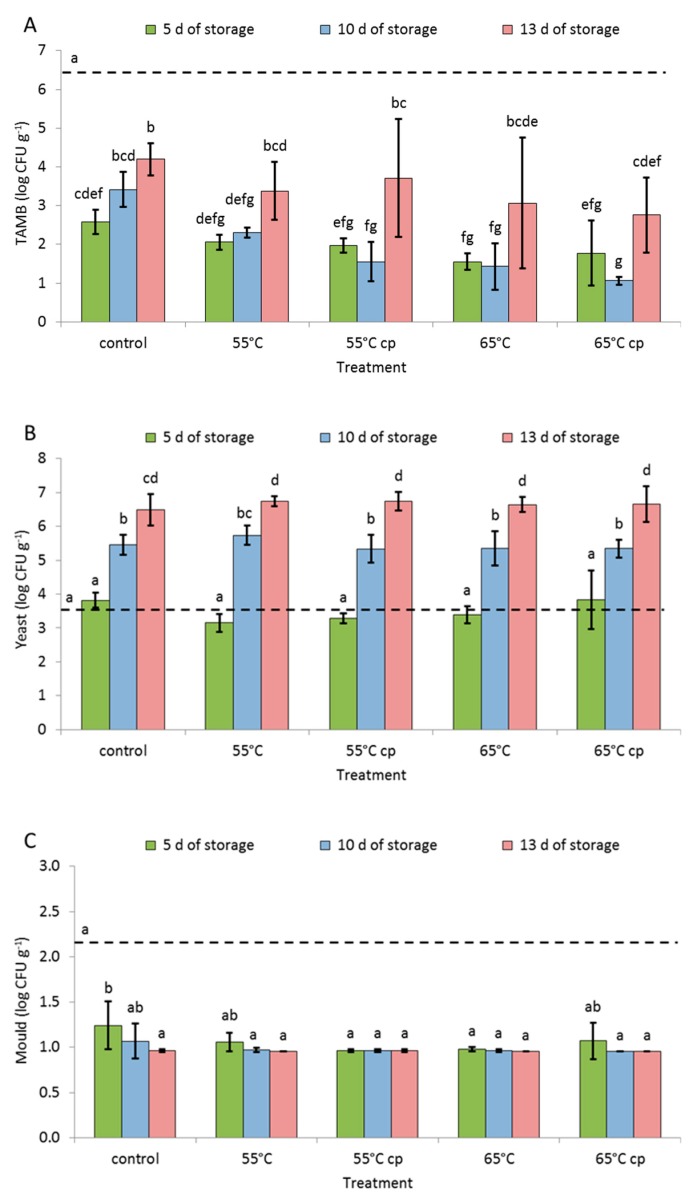
Microbial analysis of fresh, short-term hot-water treated and untreated fresh-cut apple slices stored at 4 °C in sugar syrup for up to 13 days. (**A**) Total aerobic mesophilic bacteria (TAMB), (**B**) yeast and (**C**) mold. The dotted lines represent the initial contamination. Given are means ± standard deviation (*n* = 3). Different letters indicate significant differences between means (*p* < 0.05).

Compared to initial total aerobic mesophilic bacteria (TAMB) counts of fresh apples (6.4 Log CFU g^−1^), TAMB counts were strongly reduced after treatments in all samples but partially increased again with prolonged storage (Figure 3A). On day 5, there was no clear cut effect on TAMB amongst samples of the different treatments, although sHWT and acid-treated apples always showed slightly smaller counts. This effect was more pronounced after 10 days but not after 13 days of storage. The initial yeast counts (3.5 Log CFU g^−1^) of fresh apples did not significantly change during early storage, but significantly increased with longer storage, irrespective of the treatment (Figure 3B). In samples of all treatments, mold counts were significantly lower after treatments (Figure 3C) than in fresh apples (initial counts: 2.2 Log CFU g^−1^). Some sHWT samples (55 cp and 65) showed significantly lower mold counts than controls. From day 10 onwards, no differences between mold counts were observed irrespective of the treatments.

**Figure 4:**
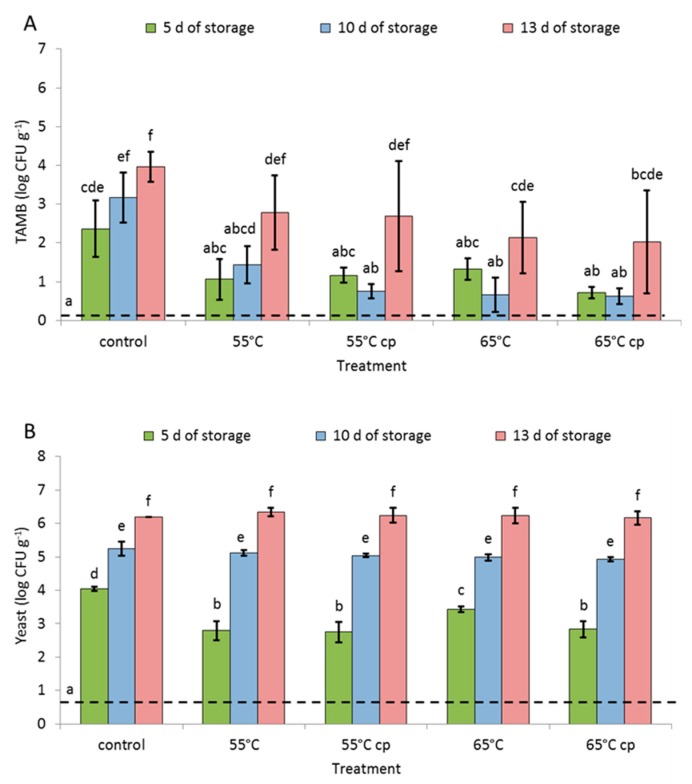
Microbial analysis of sugar syrup containing fresh, short-term hot-water treated or untreated fresh-cut apple slices stored at 4 °C for up to 13 days. (**A**) Total aerobic mesophilic bacteria (TAMB) and (**B**) yeast. The dotted lines represent the initial contamination. Given are means ± standard deviation (*n* = 3). Different letters indicate significant differences between means (*p* < 0.05).

The TAMB in sugar syrup increased during storage irrespective of the treatments (Figure 4A). Except for controls, this increase, however, was not significant. The TAMB load of the sugar syrup of apple slices treated at 65 °C and, additionally, with acid solution (65 cp) was significantly lower than that of the sugar syrup of controls. No significant growth of TAMB was observed in sugar syrup until day 10 of storage. However, syrup containing samples treated at 55 °C plus acid (55 cp) and at 65 °C without acid (65) had significant lower TAMB loads on day 10 if compared to that of controls. After prolonged storage, TAMB counts were significantly higher in control syrup and that of 55 °C sHWT samples, if compared to that on day 5.

No molds could be detected in sugar syrups, irrespective of treatments and time of storage. In contrast, the low yeasts counts in clean sugar syrups (0.7 Log CFU g^−1^) continued to significantly increase with storage for all syrups (Figure 4B). Only on day 5, the sugar syrups that had contained sHWT samples showed significantly lower yeast counts than those containing controls. However, sugar syrups of 65 °C-treated apple slices yielded higher yeast counts than those of the other sHWT samples.

## 4. Discussion

### 4.1. Tissue and Peel Color

Surface color is a very important quality parameter of fresh-cut fruit, which greatly impacts consumers’ acceptance [28]. In particular, extensive surface browning of cut tissues may lead to the rejection of fresh-cut fruit salads.

In the present study, storage in the commercial sugar syrup, regularly containing ascorbic acid, sodium ascorbate and citric acid, effectively prevented tissue browning (indicated by the browning index, BI) in all samples. The additional acid treatments after cutting did not further enhance this and appeared to have no advantageous effect. Furthermore, sHWT did not significantly affect tissue color of apple slices.

Tissue browning is closely related to the concentration of phenolic compounds and to the activity of the key enzyme polyphenol oxidase [29], while tissue pH, temperature and especially the availability of oxygen also play important roles [29]. Consequently, reduced O_2_ concentrations may prevent browning and, thus, the reduction of lightness L* as, e.g., shown for ‘Golden Delicious’ apple slices in 100% N_2_ [30]. Storage of apple slices in modified atmosphere packaging (MAP) at 0–1 kPa O_2_ [31] or at 5 kPa O_2_ + 5 kPa CO_2_ [32] may even increase tissue lightness. Furthermore, storing fresh-cut apple slices immerged in commercial sugar syrups of different sugar concentrations completely prevented surface browning [6]. This effect was attributed to both the reduced oxygen availability in the syrup [6] and to the addition of citric acid as active anti-browning agent [33,34] to the sugar solution. The reduced pH in the syrup may further intensify the inhibition of enzymatic browning [35,36,37].

In the present study, peel browning was not observed in any treatment. Apple slices treated at 65 °C, however, experienced a slightly reduction of the red peel color due to the degradation of dissolved anthocyanins, while the yellow color increased compared to controls. This might be mainly attributed to the fact that, in contrast to the cells of the fruit body tissue, the epidermis and few hypodermal cell layers were pronouncedly heated [26]. Treating apples at temperatures above 55 °C may cause necrotic damages and browning of the apple peel [18]; whereas temperatures below 50 °C even prevented enzymatic browning due to inhibition of the polyphenol oxidases [38,39].

### 4.2. Tissue Strength

Tissue strength is a very complex parameter [40] resulting from multiple tissue and cell properties such as tissue structure, cell to cell adhesion, turgor, cell size, and biochemical and biophysical characteristics of cell walls [28,41]. Tissue softening can be related to various physiological processes such as hydrolysis of protopectins to water-soluble pectins, diffusion of symplastic sugars into intercellular spaces, decrease in cellulose crystallinity, movement of ions out of the cell wall, or cell wall thinning [28]. In the present study, tissue strength significantly and more or less continuously declined during storage in sugar syrup. At the relatively mild temperature and the short incubation time used, sHWT did not accelerate softening but tended to diminish this effect. These findings are supported by earlier reports that heat-treatment may improve tissue strength of intact apples [20,42]. Inhibition of softening by sHWT was in particular obvious in samples not additionally treated with acid solutions. In this context, Ponting et al. [43] and Rojas-Graü et al. [9] also reported a decrease in the tissue strength in ascorbic acid treated apple slices. It can, thus, been assumed that these treatments may induce or facilitate acid related degradation of pectins [44] and, concomitant, cell wall weakening, thus leading to softening. Rux et al. [6], investigating the effects of different immersion media on quality properties of fresh-cut apple slices, indicated that the osmolality of syrups had the strongest influence on tissue softening. Immersion of samples in 20% sugar solutions even slightly (9%) enhanced tissue strength compared to fresh-cut samples. Nevertheless, in all the above mentioned studies, the choice of cultivars [38] and the stage of fruit maturity may certainly also play important roles [9,45].

### 4.3. Soluble Solid Content, Total Titratable Acidity and Vitamin C

Results of the present study clearly highlighted that neither sHWT temperature nor storage in sugar syrup affected the sugar contents (as indicated by TSS) of apple slices. This coincides with other investigations showing that storage of fresh-cut apple slices in air (6 d; [6]) or in low-oxygen atmosphere (at 4 °C for 12 d; [32]) did not significantly change the TSS of samples. On the other hand, Biegańska-Marecik and Czapski [46] reported that pre-treating fresh-cut apple slices with different sucrose solutions before vacuum packing increased the TSS of samples depending on the respective sugar concentrations. In apple slices, stored in sugar solutions of various concentrations, the TSS gradually decreased (5–10%) or increased (>20%) according to the osmotic content of each solution [6]. Thus, the concentration of sugar syrup (approx. 20%) used in the present study was optimal and did not affect the sugar content, a very important component of fruit taste.

On the other hand, the sHWT at both temperatures had no (negative) effects on TSS, total titratable acids or vitamin C contents of fresh-cut apple slices. The increase in TA and in vitamin C observed during storage reflects the absorption of the certain amounts of ascorbic and citric acid, regularly added to the commercial sugar syrup. This is, in particular, obvious for the vitamin C contents of samples, additionally treated with the acid solution before storage. In this context, the ascorbic acid content of this acid solution is more than four times that of the commercial sugar syrup (see Materials and Methods). Similarly, Cocci et al. [33] reported a significant increase in TA in apple slices after immersion in antioxidant solution (3 min, 1% ascorbic acid + 1% citric acid).

### 4.4. Microbial Load

In this study, sHWT and post-processing acid treatment, two approaches to reduce microbial contaminations, showed to work successfully for fresh-cut apple samples. Generally, the reduction mechanism of the post-cutting acid treatment is related to the antimicrobial effects of high acidity. This is the most important reason for the practical use of this approach. However, both techniques are not synergistic but resulted in similar reduction effects. Nevertheless, this also indicates the capability of sHWT to replace the chemical sanitation method. The processing industry is very interested in applying sHWT because it can lower the use of chemicals and may reduce production costs, although the energy expenditure must be considered.

In HWT, temperatures between 50 and 55 °C are generally assumed to effectively inactivate bacteria and yeasts [20,47,48], the dominant microorganisms on intact apples [18,49]. Spadoni et al. [21] e.g., reported an 80% reduction of total bacterial counts by HWT in the above mentioned temperature range. HWT of apples at 55 °C for 0.5 to 2 min successfully reduced inoculated bacteria, yeasts and molds by 2–3 log.

In the present study, all treatments applied pronouncedly reduced the initially high TAMB load of fresh apples during early storage. Although sHWT sustainably lowered TAMB counts both on apple slices and in the syrup, a major effect may be ascribed to the acid treatment [6]. On the other hand, immersion of apple slices in sugar syrup alone did not reduce microbial loads. Furthermore, sHWT at the higher temperature of 65 °C or the combination of both acid and sHWT only marginally improved hygienisation effects. Furthermore, sHWT of 65 °C appeared to shorten the shelf life of intact apples [18].

Yeast counts recorded for fresh apples and for apple slices after 5 days of storage in this study closely reflected results recently reported for intact apples [18]. Although not obvious from the present results, acid- and/or sHWT presumably immediately reduced yeast counts, but continuous yeast growth led to identical counts on all treated samples after 5 days of storage. This duration of shelf life corresponds to the commonly demanded minimum shelf life for fresh-cut fruit salads [15]. Only yeast counts exceeded the limits (5 log CFU g^−1^) defined by the German Association for Hygiene and Microbiology (DGHM) [50] for fresh-cut and packaged fruits after 10 days of storage. Therefore, in the present study, yeasts are most relevant for the maximum obtainable shelf life of fresh and fresh-cut apples immersed in sugar syrup. In case of yeasts, however, sHWT did only indirectly and temporally extended shelf life of fresh-cut fruit salads.

In this context, molds did not cause any problem. Although sHWT somewhat intensified the positive effects, mold counts were reduced after all treatments and showed no further growth. This was probably due to the low pH combined with the high sugar contents in the syrup, which effectively suppressed any mold growth [51].

The fact that overall microbial loads were not lower on slices of sHWT apples than on post-cutting acid treated controls may rule out the induction of protective physiological heat acclimation processes [26]. The above results, in particular the lack of any effect of the temperature rise from 55 °C to 65 °C, also substantiate the assumption that a direct heat inactivation of microorganisms is not the main mechanism of HWT sanitation [26]. On the other hand, in intact fruit, this temperature increase resulted in a sustained significant growth reduction of microbes during subsequent storage. These divergent responses of intact and sliced apples may indicate that the short-term and temporary melting of the epidermal wax layer, which produces a physical barrier to subsequent infections, may play an important role as also proposed by Kabelitz et al. [26].

The microbial quality of storage syrups obviously also played a major role in the maintenance of quality and safety, because the sugar solutions presented a large proportion of the total mass (usually approx. 1/3) of stored fresh-cut fruit salads. In nearly aseptic pure syrups, all microbial counts were clearly below detection limits (<1 Log). However, syrups may be contaminated by microorganisms adherent to the slices. Under sub-optimal storage conditions, particularly yeasts grow faster in the syrup than on apple slices, where they only colonize the surface. Thus, yeast counts in the entire pail may exceed legal limits, while that on apple slices may remain acceptable.

Due to the low pH in the solution, only TAMB and yeast counts increased during storage. Interestingly, sHWT of apples significantly inhibited the increase of yeast counts in the syrup during the initial 5 days of storage, although it had no effects on yeasts on fruit slices. In addition, yeast counts exceeded DGHM limits (5 log) in the sugar syrup on day 10 and thus proved their particular relevance in spoiling of fresh-cut fruit salads stored immersed in sugar syrup.

## 5. Conclusions

Pre-processing sHWT at both 55 °C and 65 °C did not adversely affect important quality parameters of fresh-cut apple slices and had no effects on TSS, total titratable acids or vitamin C contents. No peel or tissue browning could be observed. Although tissue strength continuously declined during storage in sugar syrup, sHWT tended to partially diminish softening. Thus, the use of sHWT eliminates the need for post-processing acid treatment. Bacteria and yeasts were the dominant microorganisms on apple slices and their counts increased during storage, both on apple slices and in the syrup. Short-term HWT significantly but only temporarily inhibited growth of TAMB and yeasts in the syrup during storage. However, sHWT could not extend the shelf life of apple slices. Increasing the treatment temperature from 55 °C to 65 °C only marginally improved TAMB reduction. In the control samples without sHWT, the observed reduction of microorganisms can be mainly ascribed to the post-cutting acid treatment. However, the combination of this acid and the sHWT did not or only minimally enhance the reduction of microbes. Moreover, the combination of treatments did not further improve quality maintenance. Yeasts are the most critical parameter for the shelf life of fresh and of fresh-cut apples immersed in sugar syrup because yeast counts exceeded accepted limits for fresh-cut and packaged fruit after 10 days of storage, both on the slices and in the syrup. The microbial quality of the syrup also plays a major role because these sugar solutions present a large proportion of the total mass of stored fresh-cut fruit salads. Summarizing, the presented results pointed out the ability of sHWT to replace actual commercial post-cutting acid preservation on fresh-cut apples.

## Figures and Tables

**Table 1:** Treatments of apples slices, fresh (initial) or stored (at 4 °C; max. 13 days) in sugar syrup with or without chemical prevention.

Treatment	sHWT	cp	Immersed in Sugar Syrup	Sampling Day
initial	no	no	no	0
control	no	yes	yes	5, 10, 13
55	55 °C	no		
55_cp	55 °C	yes		
65	65 °C	no		
65_cp	65 °C	yes		

sHWT = short-term hot-water treatment; cp = chemical prevention with organic acids.

**Table 2:** Tissue color parameters (browning index (BI) and CIE L*, a*, b*) of short-term hot-water treated and untreated fresh-cut apple slices stored at 4 °C in sugar syrup for up to 13 days.

day	Treatment	BI	L*	a*	b*
0	initial	35.3 ± 6.0 (a)	67.0 ± 1.7 (a)	2.8 ± 1.1 (a)	18.8 ± 2.5 (a)
5	control	32.5 ± 1.0 (a)	63.9 ± 0.4 (bc)	2.3 ± 0.2 (ab)	16.7 ± 0.2 (b)
	55	33.2 ± 1.8 (a)	65.1 ± 1.1 (b)	1.8 ± 0.0 (b)	17.7 ± 0.9 (ab)
	55_cp	35.0 ± 1.5 (a)	63.3 ± 0.8 (bc)	2.2 ± 0.3 (ab)	17.7 ± 0.5 (ab)
	65	34.3 ± 3.5 (a)	64.5 ± 0.7 (bc)	2.1 ± 0.3 (ab)	17.8 ± 1.4 (ab)
	65_cp	32.8 ± 0.9 (a)	64.5 ± 0.7 (bc)	2.1 ± 0.2 (ab)	17.2 ± 0.3 (ab)
10	control	31.2 ± 0.6 (a)	64.0 ± 0.1 (bc)	2.2 ± 0.3 (ab)	16.2 ± 0.2 (b)
	55	32.4 ± 1.6 (a)	64.6 ± 1.1 (bc)	2.2 ± 0.6 (ab)	17.0 ± 0.1 (ab)
	55_cp	31.7 ± 1.3 (a)	64.3 ± 1.2 (bc)	2.1 ± 0.1 (ab)	16.6 ± 0.3 (b)
	65	34.1 ± 3.6 (a)	64.7 ± 1.3 (bc)	2.5 ± 0.5 (ab)	17.6 ± 1.1 (ab)
	65_cp	33.7 ± 0.7 (a)	62.8 ± 1.0 (c)	2.4 ± 0.1 (ab)	16.9 ± 0.2 (ab)
13	control	33.2 ± 0.9 (a)	63.7 ± 0.5 (bc)	2.3 ± 0.2 (ab)	17.0 ± 0.6 (ab)
	55	33.6 ± 1.4 (a)	65.2 ± 1.4 (b)	2.3 ± 0.3 (ab)	17.6 ± 0.2 (ab)
	55_cp	32.8 ± 1.8 (a)	63.9 ± 1.4 (bc)	2.2 ± 0.1 (ab)	16.9 ± 1.1 (ab)
	65	34.7 ± 1.9 (a)	64.4 ± 0.6 (bc)	2.2 ± 0.2 (ab)	18.0 ± 1.1 (ab)
	65_cp	32.9 ± 1.6 (a)	64.3 ± 1.2 (bc)	2.2 ± 0.3 (ab)	17.1 ± 1.2 (ab)

Given are means (*n* = 3) ± standard deviation. Different letters indicate significant difference between means (*p* < 0.05). cp = chemical prevention with organic acids.

**Table 3:** Peel color parameters (BI and CIE L*, a*, b*) of short-term hot-water treated and untreated fresh-cut apple slices stored at 4 °C in sugar syrup for up to 13 d.

day	Treatment	BI	L*	a*	b*
0	initial	60.7 ± 6.3 (abc)	52.9 ± 2.5 (abc)	17.1 ± 3.3 (a)	16.6 ± 1.8 (a)
5	control	63.0 ± 2.1 (abcd)	53.0 ± 4.4 (abc)	13.9 ± 2.5 (ab)	19.1 ± 3.7 (ab)
	55	65.6 ± 1.7 (abcde)	55.6 ± 2.5 (bc)	12.4 ± 2.0 (bc)	21.9 ± 2.8 (bc)
	55_cp	61.8 ± 1.9 (abcd)	55.6 ± 2.1 (abc)	10.3 ± 1.4 (bcd)	21.5 ± 0.9 (bc)
	65	69.8 ± 4.7 (de)	57.3 ± 2.2 (c)	9.8 ± 3.3 (bcd)	25.3 ± 4.1 (cd)
	65_cp	65.5 ± 2.7 (abcde)	55.3 ± 0.5 (bc)	11.2 ± 0.7 (bcd)	22.3 ± 1.1 (bc)
10	control	59.3 ± 6.3 (ab)	55.0 ± 2.3 (ab)	12.0 ± 3.1 (bc)	19.5 ± 0.7 (ab)
	55	61.7 ± 3.7 (abcd)	56.5 ± 1.5 (abc)	10.2 ± 0.8 (bcd)	21.9 ± 1.4 (bc)
	55_cp	57.4 ± 4.6 (a)	58.6 ± 2.9 (a)	7.4 ± 2.5 (d)	22.7 ± 3.2 (bc)
	65	68.1 ± 7.3 (de)	57.7 ± 3.1 (c)	8.5 ± 2.4 (cd)	25.4 ± 0.6 (cd)
	65_cp	64.8 ± 5.4 (abcde)	57.0 ± 2.0 (abc)	8.2 ± 2.2 (cd)	24.2 ± 2.8 (cd)
13	control	65.3 ± 3.4 (abcde)	55.8 ± 2.6 (bc)	12.2 ± 2.0 (bc)	21.8 ± 1.5 (bc)
	55	66.6 ± 1.8 (bcde)	58.0 ± 1.0 (c)	8.8 ± 2.2 (cd)	25.0 ± 1.8 (cd)
	55_cp	63.8 ± 1.7 (abcde)	58.9 ± 1.2 (abc)	6.8 ± 2.3 (d)	25.4 ± 1.8 (cd)
	65	72.1 ± 3.1 (e)	57.9 ± 1.2 (c)	7.4 ± 0.8 (d)	27.4 ± 1.8 (d)
	65_cp	67.8 ± 2.1 (cde)	57.3 ± 3.0 (c)	9.3 ± 2.5 (cd)	24.9 ± 3.2 (cd)

Given are means (*n* = 3) ± standard deviation. Different letters indicate significant difference between means (*p* < 0.05). cp = chemical prevention with organic acids.
